# Endophilin A1 Promotes Actin Polymerization in Dendritic Spines Required for Synaptic Potentiation

**DOI:** 10.3389/fnmol.2018.00177

**Published:** 2018-05-28

**Authors:** Yanrui Yang, Jiang Chen, Zhenzhen Guo, Shikun Deng, Xiangyang Du, Shaoxia Zhu, Chang Ye, Yun S. Shi, Jia-Jia Liu

**Affiliations:** ^1^State Key Laboratory of Molecular Developmental Biology, Institute of Genetics and Developmental Biology, Chinese Academy of Sciences, Beijing, China; ^2^CAS Center for Excellence in Brain Science and Intelligence Technology, Chinese Academy of Sciences, Shanghai, China; ^3^State Key Laboratory of Pharmaceutical Biotechnology and MOE Key Laboratory of Model Animal for Disease Study, Model Animal Research Center, Nanjing University, Nanjing, China; ^4^Graduate School, University of Chinese Academy of Sciences, Beijing, China; ^5^College of Life Sciences, University of Chinese Academy of Sciences, Beijing, China

**Keywords:** endophilin A1, learning and memory, synaptic transmission, synaptic potentiation, actin polymerization, structural plasticity, dendritic spine, AMPAR

## Abstract

Endophilin A1 is a member of the N-BAR domain-containing endophilin A protein family that is involved in membrane dynamics and trafficking. At the presynaptic terminal, endophilin As participate in synaptic vesicle recycling and autophagosome formation. By gene knockout studies, here we report that postsynaptic endophilin A1 functions in synaptic plasticity. Ablation of endophilin A1 in the hippocampal CA1 region of mature mouse brain impairs long-term spatial and contextual fear memory. Its loss in CA1 neurons postsynaptic of the Schaffer collateral pathway causes impairment in their AMPA-type glutamate receptor-mediated synaptic transmission and long-term potentiation. In KO neurons, defects in the structural and functional plasticity of dendritic spines can be rescued by overexpression of endophilin A1 but not A2 or A3. Further, endophilin A1 promotes actin polymerization in dendritic spines during synaptic potentiation. These findings reveal a physiological role of endophilin A1 distinct from that of other endophilin As at the postsynaptic site.

## Introduction

**FIGURE 1:**
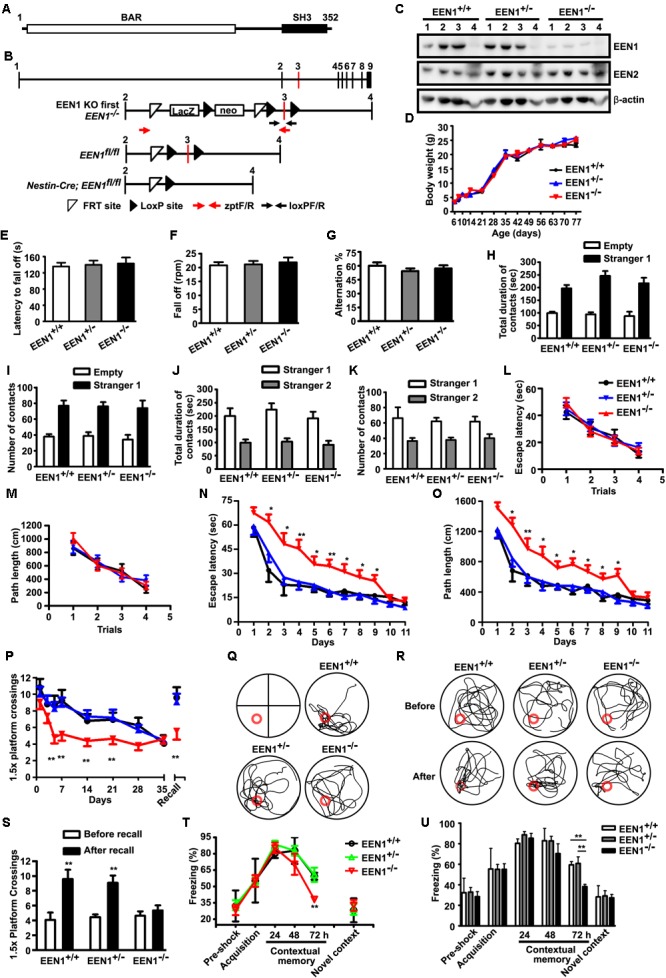
EEN1 KO mice are impaired in long-term spatial and contextual fear memory. **(A)** Domain structure of EEN1. **(B)** Schematic representation of the EEN1 gene locus, the KO-first, floxed and mutant alleles after homologous recombination. zptF/R and loxPF/R: primer pairs used for genotyping. neo, the neomycin resistance cassette. **(C)** Immunoblots of tissue lysates from mouse littermates, probed with antibodies to EEN1 and EEN2. β-Actin serves as loading control. 1, hippocampus; 2, cortex; 3, cerebellum; 4, liver. **(D)** No differences in the body weight of *EEN1^+/+^, EEN1^+/-^*, and *EEN1^-/-^* mice were detected during development (9 *EEN1^+/+^*, 11 *EEN1^+/-^*, and 14 *EEN1^-/-^*). **(E–G)** No effects of *EEN1* KO on the performance in assays of rotarod **(E** and **F)** and Y maze **(G)**. Data represent mean ± SEM for each group (18 *EEN1^+/+^*, 26 *EEN1^+/-^*, and 22 *EEN1^-/-^*). **(H–K)** No effects of *EEN1* KO on the social affiliation and sociability **(H** and **I)** or social memory and novelty **(J** and **K)**. Data represent mean ± SEM (9 *EEN1^+/+^*, 10 *EEN1^+/-^*, and 8 *EEN1^-/-^*). **(L–S)** The Morris water maze test. Shown are escape latency or traveled distance before escaping to the platform among groups in the visible-platform training **(L** and **M)**, escape latency, and traveled distance before escaping to the platform in the invisible-platform training **(N** and **O)**, number of crossing with the 1.5× platform area over 35 days after training and the swim trace 7 days after training in the probe test **(P** and **Q)**, the swim trace and recall ability following training once again on day 35 **(R** and **S)**. Red circle indicates position of the platform. Data represent mean ± SEM (9 *EEN1^+/+^*, 10 *EEN1^+/-^*, and 8 *EEN1^-/-^*), ^∗^*p* < 0.05, ^∗∗^*p* < 0.01. **(T** and **U)** Contextual fear conditioning. Shown are levels of freezing behavior after 24, 48, and 72 h from contextual fear training, and levels of freezing when animals were exposed to a novel context. Data represent mean ± SEM (9 *EEN1^+/+^*, 11 *EEN1^+/-^*, and 10 *EEN1^-/-^*), ^∗∗^*p* < 0.01

Endophilin A1 (or endophilin 1, EEN1) is a member of the evolutionarily conserved endophilin A family that is expressed almost exclusively in brain (de Heuvel et al., 1997; Ringstad et al., 1997, 2001), featuring an amino-terminal amphipathic helix-Bin/amphiphysin/Rvs (N-BAR) domain with membrane bending and curvature sensing capacities (Farsad et al., 2001; Gallop et al., 2006; Frost et al., 2009; Bai et al., 2010), and a carboxyl-terminal Src Homology 3 (SH3) domain that binds to a number of protein partners (**Figure 1A**; Gad et al., 2000; Vinatier et al., 2006; Nakano-Kobayashi et al., 2009; Fu et al., 2011; Pechstein et al., 2015; Yang et al., 2015). Previous studies have established roles for endophilin As in recycling of synaptic vesicles through its interaction with the endocytic proteins synaptojanin, dynamin, and intersectin (Verstreken et al., 2002, 2003; Schuske et al., 2003; Milosevic et al., 2011; Pechstein et al., 2015), and regulation of neurotransmitter exocytosis through its binding to the glutamate transporter VGLUT1 (Weston et al., 2011). In mammalian cells, they also mark and control a clathrin-independent fast endocytic pathway of transmembrane receptors (Boucrot et al., 2015; Renard et al., 2015). Most recently endophilin As were found to be involved in autophagosome formation and protein homeostasis at presynaptic terminals of neuromuscular junctions (NMJ) in *Drosophila* and mammalian neurons (Murdoch et al., 2016; Soukup et al., 2016).

In hippocampal neurons, all three members of endophilin A family localize to both pre- and post-synaptic sites (Chowdhury et al., 2006; Yang et al., 2015). Although knockout (KO) of individual endophilin A genes in mice does not affect life span and fertility, double knockout (DKO) of endophilin A1 and A2 (or endophilin 2, EEN2) genes causes progressive ataxia and neurodegeneration, and triple knockout (TKO) causes perinatal lethality (Milosevic et al., 2011; Murdoch et al., 2016), suggesting functional redundancy among them in neurons. At the presynaptic terminal, DKO or TKO causes accumulation of clathrin-coated vesicles and impairment in synaptic transmission (Milosevic et al., 2011). Intriguingly, although cell biological studies and electron microscopy analysis of DKO and TKO synapses reveal a role of endophilin As in clathrin uncoating after scission of endocytosed synaptic vesicles at the presynaptic site, a decrease in the amplitude of spontaneous miniature excitatory postsynaptic currents (mEPSC) was detected in TKO neurons (Milosevic et al., 2011), implying changes in the number of the AMPA-type glutamate receptors (AMPARs) in postsynaptic plasma membrane that cannot be explained by their known functions.

At the postsynaptic site, endophilin A2 and A3 (or endophilin 3, EEN3) interact with the immediate early protein Arc/Arg3.1 to enhance endocytic trafficking of the AMPARs that likely contributes to synaptic plasticity and memory consolidation (Chowdhury et al., 2006; Rial Verde et al., 2006). In dendritic spines, membrane protrusions from dendrites that are major postsynaptic sites for excitatory inputs, endophilin A1 interacts with the cytoskeleton regulator p140Cap and regulates spine morphogenesis and synapse formation during early neurodevelopment (Yang et al., 2015). Whether or not postsynaptic endophilin A1 also functions in synaptic plasticity is unclear. Moreover, the physiological function(s) of individual endophilin As in the mammalian central nervous system (CNS) remain elusive.

In this study, we have investigated the postsynaptic function(s) of endophilin A1 using single gene KO mice and mature mouse hippocampal neurons. Endophilin A1 KO mice exhibit significantly impaired contextual fear memory and spatial learning and memory. Hippocampal CA1-specific KO of endophilin A1 in adult animals causes similar memory phenotypes to those of whole brain KO, indicating that its function in the hippocampus CA1 is required for long-term memory. Moreover, ablation of endophilin A1 in CA1 neurons impairs AMPAR-mediated synaptic transmission and long-term potentiation (LTP). We further show that endophilin A1 promotes actin polymerization required for the morphological and functional changes in dendritic spines of cultured hippocampal neurons during chemically induced LTP. These findings uncover a postsynaptic role of endophilin A1 in synaptic plasticity and long-term memory.

## Materials and Methods

### Ethics Statement

All animal experiments were approved (approval code AP2013003 and AP2015002) by the Animal Care and Use Committee of the Institute of Genetics and Developmental Biology, Chinese Academy of Sciences. The *Nestin-Cre-Tg* C57BL/6J mice were obtained from Nanjing Biomedical Institution of Nanjing University (Tronche et al., 1999). The *Thy1-EGFP-Tg* C57BL/6J mice were obtained from the Jackson Laboratory (Feng et al., 2000). All animals were housed in standard mouse cages at 22–24°C on a 12 h light/dark cycle with access to food and water freely.

### Generation of Endophilin A1 Knockout Mice

The targeting vector for *EEN1* was obtained from European Mouse Mutant Cell Repository (EuMMCR, PRPGS00060_A_A02). The endophilin A1 KO first and *EEN1*^fl/fl^ C57BL/6J mice were generated at Nanjing Biomedical Institution of Nanjing University. *EEN1* CNS-specific KOs were generated by crossing *EEN1*^fl/fl^ mice with *Nestin-Cre-Tg* mice. Mice with a limited subset of green fluorescent protein (GFP)-labeled neurons for analysis of spine morphology were generated by crossing *Nestin-Cre^+/-^*; *EEN1*^fl/fl^ mice to *Thy1-EGFP^+/-^*; *EEN1*^fl/fl^ mice. Genotyping of mouse lines was performed by genomic PCR. PCR genotyping of tail prep DNA from offspring was performed with the following primer pairs:

loxPF/loxPR: 5′-CAAGGACTCCCAGAGACCTAGCATC-3′ and 5′-GAGATGGCGCAACGCAATTAAT-3′ [PCR primer locations are shown in **Figure 1B** resulting in a PCR product of 375 base pairs in EEN1 KO first mice but none in wild-type (WT) mice].

zptF/zptR: 5′-GTAAGCGGCTCTAGCGCATGTTCT-3′ and 5′-GCAGGGGCATGTAGGTGGCTCAAC-3′ (PCR primer locations are shown in **Figure 1B**. Genomic PCR results in a PCR product of 466 base pairs in WT mice, none in EEN1 KO first mice, and of 627 base pairs in *EEN1*^fl/fl^ mice).

The *Nestin-Cre* transgene was detected using the following primer pairs:

5′-TGCCACGACCAAGTGACAGCAATG-3′ and 5′-ACCAGAGAGACGGAAATCCATCGCTC-3′.

The *Thy1-EGFP* transgene was detected using the following primer pairs:

5′-TCTGAGTGGCAAAGGACCTTAGG-3′ and 5′-CGCTGAACTTGTGGCCGTTTACG-3′.

### Constructs, Viruses, and Stereotaxic Injection

The pAOV-CaMKIIα-EGFP-2A-EEN1 construct was generated by cloning EEN1 cDNA amplified from pCMV-Tag2B-EEN1 into pAOV-CaMKIIα-EGFP-2A. All other constructs used in this study (EEN1-LentiGFP, pCMV-Tag2B-EEN1, pCMV-Tag2B-p140Cap, pCMV-Tag2B-EEN1 Y343A, and LifeAct-mCherry) were described previously (Yang et al., 2015). Viral particles of adeno-associated virus (AAV) carrying pAOV-CaMKIIα-EGFP-2A-Cre, pAOV-CaMKIIα-EGFP-2A-EEN1, or the control construct pAOV-CaMKIIα-EGFP-2A-3FLAG were purchased from Obio Technology (Shanghai) Corp. Ltd. (Shanghai, China).

For viral injection, 8-week-old mice were anesthetized with isoflurane (1–2% mixed with oxygen) and placed in a stereotaxic apparatus. After being sterilized with iodophors and 75% (vol/vol) alcohol, the scalp was incised along the midline between the ears. Holes were drilled in the bilateral skull. The coordinates of viral injection relative to bregma were as follows: 2.0 mm posterior, 1.8 mm lateral, and 1.4 mm ventral. Using a microinjection system (World Precision Instruments), viral particles carrying pAOV-CaMKIIα-EGFP-2A-Cre, pAOV-CaMKIIα-EGFP-2A-EEN1, or vector (1 μl, 2.0 × 10^12^ viral genomes/ml) were injected in the hippocampal CA1 region at a rate of 0.125 μl/min, the needle was kept in place for 5 min before withdrawal, the skin was sutured, and the mice were placed beside a heater for recovery (Barbash et al., 2013).

### Antibodies

The following antibodies were obtained from commercial sources: goat anti-endophilin A1 (S-20) and endophilin A2 (E-15), mouse anti-synaptophysin (SYP) (D-4) and mouse anti-cortactin (sc-55588) from Santa Cruz Biotechnology (Santa Cruz, CA, United States); rabbit anti-endophilin A1 (Synaptic Systems GmbH, Germany); rabbit and mouse anti-GFP (MBL598, D153-3), rabbit and mouse anti-RFP (PM005 and M155-3) which recognize DsRed and mCherry from Medical & Biological Laboratories (Naka-ku, Nagoya, Japan); mouse anti-MAP2 (MAB3418, Chemicon, CA, United States); mouse anti-PSD95 (75-028) for immunofluorescence staining (NeuroMab, Davis, CA, United States); mouse anti-PSD95 for western blotting (BD Biosciences, San Diego, CA, United States); mouse anti-GluA1 (MAB2263, Millipore, Billerica, MA, United States); rabbit anti-FLAG M2 (F7425), mouse anti-α-tubulin (T9026), and mouse anti-β-actin (A5441) (Sigma–Aldrich, St. Louis, MO, United States). Rabbit anti-p140Cap was described previously (Yang et al., 2015). Secondary antibodies for immunofluorescence staining were from Molecular Probes (Invitrogen, Carlsbad, CA, United States).

### Histology

Adult mice were anesthetized with 1% sodium pentobarbital and transcardially perfused with normal saline followed by 4% paraformaldehyde (PFA) in 0.01 M phosphate-buffered saline (PBS). Mouse brain was dissected out and post-fixed with 4% PFA/PBS for 4 h at 4°C. Fixed brain was incubated with PBS + 20% sucrose overnight and then PBS + 30% sucrose overnight. The brain was stored at -80°C until usage. Thirty-micron cyrosections were cut using cryostat and mounted on the slide-glass for immunostaining.

For LacZ staining, slide-glass was incubated with 1 mg/ml X-gal in the staining buffer supplemented with 5 mM potassium ferricyanide and 5 mM potassium ferrocyanide overnight at 37°C. Stained samples were washed with PBS three times then dehydrated in ethanol of ascending purity (50, 75, 90, and 100%, 2 min each). Slides were mounted on Permount and stored at room temperature (RT) (Kokubu and Lim, 2014).

For immunostaining of brain sections, floating 30-μm-thick slices were rinsed with PBS and permeabilized in 0.4% Triton X-100 in 0.01 M PBS. Cyrosections were blocked with 1% BSA in PBS containing 0.4% Triton X-100 for 1 h at RT, then incubated with primary antibodies overnight at 4°C. Appropriate secondary antibodies conjugated with Alexa Fluor 488, Alexa Fluor 555, or Alexa Fluor 647 were used for detection. Sections were then incubated with DAPI for nuclear staining for 1 h at RT. Following rinsing, cyrosections were mounted on gelatin-coated slides and covered with coverslip with mounting medium. Confocal images were collected using the Spectral Imaging Confocal Microscope Digital Eclipse C1Si (Nikon, Tokyo, Japan) with a 10× Plan Apochromat DIC N1 0.45 objective or 40× Plan Apochromat VC NA 1.40 oil objective (Yang et al., 2015).

### Behavioral Analyses

Ten-week-old male animals were used for behavioral analyses.

### Rotarod

Motor coordination and balance were assayed with an accelerating rotarod (Ugo Basile, Italy). Mice were placed on a slowly rotating drum for 1 min at 4 rpm for three times to habituate, then the rod accelerated gradually from 4 to 40 rpm over a period of 5 min. The latency and the velocity to fall off the rod were recorded.

### Y-Maze Spontaneous Alternation

The Y-maze apparatus is made of three identical arms at 120° angle with respect to each other. Mouse was put in the center of the maze and allowed to freely explore its three arms for 6 min. Alternations were defined as successive entries into each of the three arms as on overlapping triplet sets (i.e., ABC, BCA, …). Percentage of spontaneous alternations was defined as the ratio of actual (= total alternations) to possible (= total arm entries-2) number of alternations × 100.

### Social Interaction

The social interaction test was based on the method described by Kaidanovich-Beilin et al. (2011). Briefly, mouse was placed into the middle chamber and habituated for 5 min. Then the walls between chambers were removed to allow the mouse to freely explore the three chambers with two empty wire containment cups placed in the middle of both side chambers for the first 10 min. Then “stranger 1” mouse was placed inside cup located in one of the side chambers for a second 10 min. For a third 10 min, “stranger 2” mouse was placed inside cup located in the opposite side chamber. Direct contact between the subject mouse and the containment cup, or stretching of the body of subject mouse in an area 3–5 cm around the cup was counted as an active contact. Duration and number of direct (active) contacts between the subject mouse and the containment cup housing or not housing the mouse for each chamber individually were monitored by a centrally placed video camera and analyzed with an automated video tracking software (the Anilab System, AniLab Software and Instruments Co., Ltd.).

### Morris Water Maze

The water maze procedure was similar to previously established protocols (Bromley-Brits et al., 2011) with minor modifications. The water tank is a 120 cm diameter circular pool. Cues with different shapes are pasted on the wall of the tank above water surface in four different directions. A circular black curtain around the tank eliminates competing environmental cues. Nontoxic white tempura paint was used to opacify the water, which was maintained at 19–23°C. For the visible trial, a flag was placed on the platform to increase its visibility, then the flag was removed and additional water was added to the pool to submerge the platform which was kept in fixed position to 1 cm below the water surface. Acquisition training was then performed for 8 or 11 days and four trials per day with different water-entering site (at north, south, east, and west positions adjacent to the pool wall). During each trial, mouse must learn to use cues to navigate a path to the hidden platform within 90 s. If they failed to locate the platform within time, they were gently guided to it, and kept on it for 10 s. The escape latency (the average value of time duration from entering water to finding the platform of four trials per day) and traveled distance were calculated for each mouse. After acquisition training, the hidden platform was removed and probe testing was performed with one trial each day for 5 days or one trial with 2 or 7 days interval for 35 days at the distal water-entering site away from the platform. A 1.5× platform circle area where the platform was placed was monitored. The number of crossing the 1.5× platform circle area of each mouse within 60 s was analyzed. For recall training, mouse was placed in the same pool without platform to examine memory extinguishment at least 1 month after training. Similarly, the numbers of crossing 1.5× area of each mouse within 60 s were analyzed. Afterward, the platform was placed back to pool and recall training was performed for 1 day with one trial at the farthest water-entering site away from the platform. The second day, the platform was removed again and mouse was placed in pool at the farthest water-entering site away from the platform. The number of crossing 1.5× area of each mouse was analyzed. The mouse trajectory in the pool was monitored and analyzed with an automated system (Smart 3.0, Panlab SMART video tracking system).

### Contextual Fear Conditioning

Mice were trained in a standard fear conditioning apparatus (Harvard Apparatus Ltd., Holliston, MA, United States). They were allowed to explore freely for 2 min. A 2 s, 0.9 mA foot shock (unconditioned stimulus) was delivered and mice stayed in the chamber for 30 s. Mice were re-exposed to the same chamber for 2 min on the second, the third, and the fourth day. After 3 h on the fourth day, mice were exposed to a novel chamber. Freezing was scored and analyzed automatically using FREEZING software (Harvard Apparatus Ltd., Holliston, MA, United States), with thresholds set to give agreement with blinded human observation.

### Electrophysiology in Slice Cultures

Hippocampi of postnatal day 6–8 (P6-8) *EEN1^fl/fl^* mice were isolated in the ice cold dissection solution [MEM (Gibco, 12360-038) with 25 mM HEPES (Gibco, 12360-038), penicillin–streptomycin (Gibco, 15140-122), and 10 mM Tris, pH 7.2]. The isolated hippocampi were sliced to 400 μm sections with tungsten filament slicer (Siskiyou, MX-TS). Sections were cultured with medium containing 50% MEM, 25% HBSS (24020-117), 25% heat-inactivated horse serum (Gibco, 16050-122), 1 mM L-glutamine (Gibco, 35050-061), 1% penicillin–streptomycin (Gibco, 15140-122), 12 μg/ml ascorbic acid, and 1 μg/ml insulin, and supported by sterile 30-mm diameter, porous (0.4 μm), transparent, and low protein-binding membrane (Millicell-CM, Millipore, Billerica, MA, United States). The slices were infected with AAV-GFP-2A-Cre for 24 h in culture. Experiments were done 2–3 weeks after AAV infection. Slices were maintained in artificial cerebrospinal fluid (ACSF, in mM, NaCl 119, KCl 2.5, NaH_2_PO_4_ 1, NaHCO_3_ 26, CaCl_2_ 2.5, MgCl_2_ 1.3, glucose 11) supplemented with 10 μM 2-chloroadenosine to dampen epileptiform activity, and GABA receptors were blocked with picrotoxin (PTX, 0.1 mM) and bicuculline (Bic, 0.01 mM), in a solution saturated with 95% O_2_/5% CO_2_. CA1 pyramidal cells were visualized by infrared differential interference contrast microscopy. The internal solution contained (in millimolar) CsMeSO_4_ 115, CsCl 20, HEPES 10, Na_3_-GTP 0.4, Na_2_-ATP 4, EGTA 0.6, QX-314 5, and spermine 0.1. Cells were recorded with 4- to 6-MΩ borosilicate glass pipettes, following stimulation of Schaffer collaterals (SC) with concentric biopolar electrode (FHC, CBBRC75) placed in stratum radiatum at the CA1 region. All paired recordings involved simultaneous whole-cell recordings from one GFP-positive neuron and one neighboring GFP-negative neuron. GFP-positive neurons were identified by epifluorescence microscopy. Series resistance was monitored and not compensated, and cells in which series resistance was above 30 MΩ or varied by 25% during recording session were discarded. Synaptic responses were collected with the Multiclamp 700B amplifier and Digidata 1550 data acquisition system (Axon Instruments), filtered at 2 kHz, digitized at 10 Hz. The stimulus was adjusted to evoke a measurable, monosynaptic EPSC in the control cell. AMPAR-mediated responses were isolated by voltage-clamping the cell at -70 mV, whereas NMDAR responses were recorded at +40 mV and amplitudes measured at 150 ms after stimulation to avoid contamination by AMPAR current.

### Electrophysiology in Acute Slices

*EEN1^fl/fl^* mice within 24 h after birth were injected with high-titer AAV stock carrying pAOV-CAMKIIα-GFP-2A-Cre (AAV-GFP-2A-Cre) (about 1 ∼ 5 × 10^13^ IU/ml). Newborns were anesthetized on ice for 2–3 min and then mounted in a custom ceramic mold before being injected with about 10 nl of viral solution at seven sites targeting the hippocampus at each cerebral hemisphere with microsyringe (Sutter Instrument) and a beveled glass injection pipette. Injected pups were returned to home cage and used for recording 2–3 weeks afterward. Transverse 350 μm hippocampal slices were cut from viral injected *EEN1^fl/fl^* mice on a Leica vibratome (VT1000 S) in high sucrose cutting solution containing (in mM): KCl 2.6, NaH_2_PO_4_ 1.25, NaHCO_3_ 26, CaCl_2_ 0.75, MgCl_2_ 7, sucrose 211, glucose 10. Freshly cut slices were placed in an incubating chamber containing ACSF, and recovered at 32°C for about 90 min before recording. The slices were perfused with ACSF containing PTX/Bic and saturated with 95% O_2_/5% CO_2_ in whole-cell LTP experiments. CA1 pyramidal cells were voltage-clamped at -70 mV and AMPAR EPSCs were evoked by stimulation at SC. LTP was induced by stimulating SC axons at 2 Hz for 90 s while clamping the cell at 0 mV, after recording a stable 3- to 5-min baseline, but no more than 6 min after breaking into the cell (Granger et al., 2013; Diaz-Alonso et al., 2017). To minimize run-up of baseline responses during LTP, cells were held cell-attached for about 1–2 min before breaking into the cell.

### Primary Neuronal Culture and Transfection

Primary neuronal cultures from hippocampi were prepared as described previously (Banker and Goslin, 1988). Briefly, Hippocampi were dissected from P0 C57BL/6J mice, dissociated with 0.125% trypsin in Hank’s balanced salt solution without Ca^2+^ and Mg^2+^ at 37°C for 20 min, triturated in DMEM, 10% F12, and 10% fetal bovine serum. Hippocampal neurons were plated on poly-D-lysine-coated coverslips in 24-well plates at a density of 2 × 10^4^ cells/well. The medium was replaced with the serum-free Neurobasal (NB) media supplemented with 2% B27 supplement and GlutaMAX (Gibco, Invitrogen, Carlsbad, CA, United States) 4 h after plating. Half of the media were changed every 3 days until use.

For neuronal morphology and immunofluorescence staining, neuronal transfections were performed using Lipofectamine LTX according to the manufacturer’s instructions (Invitrogen, Carlsbad, CA, United States) on 12–14 days *in vitro* (DIV) after plating. Briefly, DNA (1.0 μg/well) was mixed with 1 μl PLUS reagent in 50 μl NB medium, then mixed with 2.0 μl Lipofectamine LTX in 50 μl NB medium, incubated for 20 min, and then added to the neurons in NB at 37°C in 5% CO_2_ for 1 h. Neurons were then rinsed with NB and incubated in the original medium at 37°C in 5% CO_2_ for 4–5 days. For co-transfection, neurons were transfected with 1.0 μg of DNA consisting of two plasmids (0.50 μg each).

### Chemical LTP Stimuli

Neurons were treated with glycine (200 μM) in Mg^2+^-free extracellular solution (mM: 125 NaCl, 2.5 KCl, 2 CaCl_2_, 5 HEPES, 33 glucose, 0.2 glycine, 0.02 bicuculline, and 0.003 strychnine, pH 7.4) for 10 min. Neurons were then kept in extracellular solution without glycine for 30 min (Park et al., 2006; Fortin et al., 2010).

### Immunofluorescence Staining, Image Acquisition, and Analysis

For surface GluA1 labeling, neurons were fixed for 7 min at RT in PBS containing 4% PFA/4% sucrose, rinsed with PBS, blocked with 10% normal goat serum in PBS for 30 min, and incubated with mouse anti-GluA1 (anti-N terminus) antibodies in PBS with 1% normal goat serum overnight at 4°C, followed with appropriate fluorescence-conjugated secondary antibodies. Neurons were then permeabilized with 0.4% Triton X-100 for 30 min at RT followed by labeling with other primary antibodies. For all other labeling, neurons were fixed in 4% PFA/4% sucrose in PBS at RT for 15 min. After blocking with 1% BSA in PBS containing 0.4% Triton X-100 for 1 h at RT, neurons were incubated with primary antibodies for 1 h at RT or overnight at 4°C, and appropriate secondary antibodies conjugated with Alexa Fluor 488, Alexa Fluor 555, or Alexa Fluor 647 were used for detection.

Confocal images were collected using the Spectral Imaging Confocal Microscope Digital Eclipse C1Si (Nikon, Tokyo, Japan) with a 100× Plan Apochromat VC NA 1.40 oil objective. Images were *z* projections of images taken at 0.15–0.2 μm step intervals. The number of planes, typically 5–7, was chosen to encompass the entire dendrite from top to bottom.

The procedure for morphometric analysis of dendritic protrusions was described previously (Yang et al., 2015). GFP or DsRed was used as a cell-fill. The GFP- or DsRed-labeled dendrites or spines were outlined manually. Maximum image projections used in measurements of spine density, spine head area, or fluorescent signal intensity were rendered with the NIS-Elements AR software (Nikon, Tokyo, Japan) from confocal z-series images. Dendritic segments 40–120 μm from the neuronal cell body were selected for analysis. To quantify enrichment of F-actin in spines, we measured the mean intensity of LifeAct–mCherry fluorescence within the center of spines and normalized each measurement with the fluorescent signal along the adjacent dendritic shaft. The GFP- or mCherry-labeled dendrites or spines were outlined manually. All quantitative analyses were done with the NIS-Elements AR software.

To examine spine number and morphology *in vivo*, spines located at the apical dendrites or basal dendrites of dorsal hippocampal CA1 and CA3 regions were imaged in 100-μm-thick coronal sections from *Thy1-EGFP-Tg* mice. Z-stack Images (0.25 μm step intervals) were captured at 100× magnification with 4× optical zoom and reconstructed by maximum projections with the NIS-Elements AR software (Nikon, Tokyo, Japan). Spines were examined over 1000 μm dendritic segments from more than 15 dendrites for each mouse from two experimental groups.

To measure changes in the density and morphology of dendritic spines, and surface levels of GluA1 in spines upon chemical LTP, the spine number, spine head area, or fluorescence intensity of GluA1 in spines of glycine-treated *EEN1^+/+^* or *EEN1^-/-^* neurons was subtracted by the average of those without glycine application.

### PSD Fractionation

Cytosol, synaptosome, synaptosomal membrane, and PSD fractions from mouse brain were prepared using a small-scale modification of the procedure previously described (Carlin et al., 1980; Cho et al., 1992; Jaworski et al., 2009). In brief, hippocampi were homogenized on ice using 20 strokes of a Teflon-glass homogenizer in 1 ml of HEPES-buffered sucrose (0.32 M sucrose, 4 mM HEPES, pH 7.4) containing freshly added protease inhibitors, then homogenized with a syringe (20–30 strokes), followed by centrifugation at 800–1000 × *g* for 10 min at 4°C to remove the pelleted nuclear fraction (P1). The supernatant (S1, a.k.a Homogenates or Total) was centrifuged at 10,000 × *g* for 15 min to yield the crude synaptosomal pellet (P2). P2 was washed once in 1 ml HEPES-buffered sucrose and lysed by hypoosmotic shock in 900 μl ice-cold 4 mM HEPES, pH 7.4 plus protease inhibitors, and homogenized by pipetting and rotating for 30 min at 4°C. The lysate was centrifuged at 25,000 × *g* for 20 min to yield supernatant (S3, crude synaptic vesicle fraction) and pellet (P3, lysed synaptosomal membrane fraction). To prepare the PSD fraction, P3 was resuspended in 900 μl of ice-cold 50 mM HEPES, pH 7.4, 2 mM EDTA, protease inhibitors, and 0.5% Triton X-100, rotated for 15 min at 4°C and centrifuged at 32,000 × *g* for 20 min to obtain the PSD pellet. PSD pellets were resuspended in 60 μl ice-cold 50 mM HEPES pH 7.4, 2 mM EDTA plus protease inhibitors.

### Western Blotting

For expression analysis, tissues were dissected from C57BL/6J mice and rinsed once in ice-cold PBS, pH 7.4. Frozen samples were homogenized in lysis buffer (50 mM Tris–Cl pH 7.4, 150 mM NaCl, 5 mM EDTA, 0.5% Triton X-100) supplemented with protease inhibitors. Twenty micrograms of protein was loaded in each lane for subsequent Western blot analysis. Immunoblots were imaged with an Epichemi3 Darkroom system (UVP BioImaging Systems, Upland, CA, United States). For densitometric analysis, immunoreactive bands were quantified using ImageJ (National Institutes of Health, Bethesda, MD, United States).

### Statistical Analysis

All data are presented as the mean ± SEM. GraphPad Prism 5 (GraphPad Software, LaJolla, CA, United States) was used for statistical analysis. For two-sample comparisons vs. controls, Student’s *t*-test was used. One-way analysis of variance with a Dunnett’s multiple-comparison or Newman–Keuls multiple comparison *hoc* test was used to evaluate statistical significance of three or more groups of samples. A *p-*value of <0.05 was considered statistically significant.

## Results

### Pan-Neural Knockout of Endophilin A1 Causes Impairment in Spatial and Contextual Fear Memory

Endophilin A1 KO mice generated by removing the first exon have normal life span and show no obvious phenotypes such as neurodegeneration (Milosevic et al., 2011), suggesting functional redundancy or compensatory effects among the endophilin A family members. To investigate physiological functions of endophilin A1 that might be distinct from that of A2 and A3, we generated a reporter KO of endophilin A1 (KO-first or KO), in which the endophilin A1 gene (*EEN1*) was inactivated by insertion of a *lacZ-Neomycin* cassette before exon 3 (**Figure 1B**). Immunoblotting analysis showed that, compared with WT littermates (*EEN1^+/+^*), endophilin A1 expression was dramatically reduced in the brain of KO mice (*EEN1^-/-^*), whereas no decrease in endophilin A2 levels was detected (**Figure 1C**). Consistent with the previous study (Milosevic et al., 2011), endophilin A1 KO mice were viable and had normal body weight with no obvious phenotypic defects (**Figure 1D**).

To investigate role(s) of endophilin A1 in brain function, we analyzed the motor coordination, working memory, social interaction, and hippocampal-dependent memory of the *EEN1^+/+^, EEN1^+/-^* (heterozygous, or HET), and *EEN1^-/-^* mice. The performance of KO mice was indistinguishable from that of the WT and HET littermates in the rotarod test of motor coordination (**Figures 1E,F**). KO mice also exhibited normal working memory in the Y-maze test (**Figure 1G**) and normal social interaction in the three-chamber test (**Figures 1H–K**). In the Morris water maze test, KO mice spent a similar latency to escape and traveled similar swimming distances before escaping onto the visible platform (**Figures 1L,M**). In the training phase, all three genotypes improved their performance with repetitive training, with WT and HET mice showing a steeper learning curve and reaching a better performance level in fewer training days (**Figures 1N,O**). After prolonged training, KO mice could catch up to the performance level of WT and HET mice (**Figures 1N,O**). In the probe trial, although all three genotypes showed similar platform crossings 24 h after training, while WT and HET mice still remembered the platform location 1 month later, KO mice forgot the platform location within 7 days (**Figures 1P,Q**). Moreover, KO mice were unable to recall the platform location after training once again (**Figures 1P,R,S**). These data indicate that endophilin A1 deficiency impairs spatial learning and long-term retention of spatial memory after training has been finished.

Next we examined the effect of endophilin A1 KO on contextual fear memory. Mice were trained to associate a particular environment with a mild foot shock and tested for fear memory. Compared with WT and HET, although KO mice displayed similar levels of freezing when they were tested 24 h after training, their freezing behavior decreased significantly at 72 h (**Figures 1T,U**). Notably, all three genotypes exhibited similar levels of freezing when exposed to a novel context (**Figures 1T,U**), indicating that short-term memory was intact in KO mice. Together these data indicate that endophilin A1 deficiency impairs long-term retention of contextual fear memory.

### Expression of Endophilin A1 in Hippocampal CA1 Is Required for Spatial and Contextual Fear Memory

**FIGURE 2:**
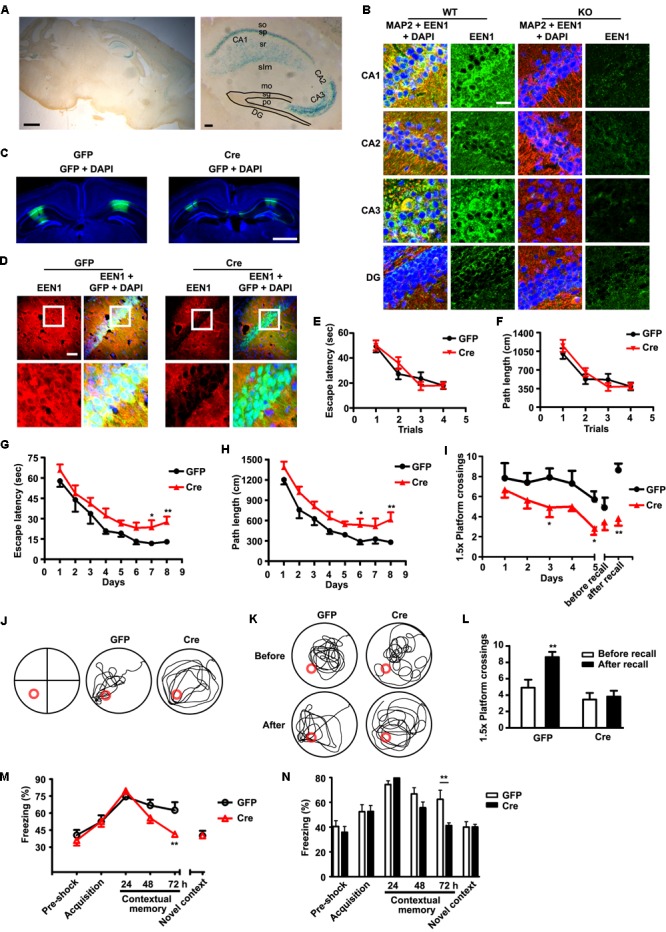
EEN1 expression in the hippocampal CA1 region of adult mice is required for long-term spatial and contextual fear memory. **(A)** LacZ staining in the sagittal brain section of 10-week-old EEN1 KO-first (*EEN1^-/-^*) mice. Right panel is magnification of the hippocampus. Scale bars, 1 mm in the left panel and 100 μm in the right panel. **(B)** Immunofluorescence staining of EEN1 in hippocampal CA1, CA2, CA3, and DG regions of 10-week-old *EEN1^+/+^* and *EEN1^-/-^* mouse brains. Scale bar, 20 μm. **(C)** AAV virus was stereotaxically injected into the CA1 regions of *EEN1^fl/fl^* mice to express GFP alone or Cre and GFP. Shown are GFP signal and DAPI labeling of nuclei 21 days after viral injection. Scale bar, 1 mm. **(D)** Immunofluorescence staining of EEN1 in brain slices 21 days after injection of AAV virus into the CA1 region of *EEN1^fl/fl^* mice. Lower panels are magnification of the boxed areas. Scale bar, 100 μm. **(E–L)** The Morris water maze test. Shown are escape latency or traveled distance before escaping to the platform in the visible-platform training **(E** and **F)**, escape latency, and traveled distance before escaping to the platform in the invisible-platform training **(G** and **H)**, number of crossing with the 1.5× platform area and the swim trace 5 days after training in probe test **(I** and **J)**, the swim trace and recall ability following training once again 1 month after training **(K** and **L)**. Data represent mean ± SEM (11 GFP, 13 Cre), ^∗^*p* < 0.05, ^∗∗^*p* < 0.01. **(M** and **N)** Decrease in freezing behavior 72 h after contextual fear training in the Cre virus-injected group. Data represent mean ± SEM (9 GFP, 12 Cre), ^∗∗^*p* < 0.01.

To investigate mechanisms underlying memory deficits caused by ablation of endophilin A1 in the CNS, first we examined its expression pattern in the brain with the endophilin A1 promoter-driven LacZ reporter (**Figure 1B**). β-Galactosidase staining of sagittal brain sections revealed enrichment of signals in CA1 and CA3 pyramidal cells in the hippocampus (**Figure 2A**). Consistently, immunofluorescence staining showed that endophilin A1 was highly expressed in hippocampal CA1 and CA3 but not in CA2 or dentate gyrus (DG) in WT mice, and its expression was dramatically downregulated in KO mice (**Figure 2B**). As both spatial memory and contextual fear memory are hippocampal-dependent functions that involve the SC-CA1 synapses (Tsien et al., 1996; Nakazawa et al., 2002; Daumas et al., 2005; Zelikowsky et al., 2012), the high expression of endophilin A1 in the hippocampus prompted us to investigate whether endophilin A1 in the CA1 region is required for spatial and contextual fear memory. To determine whether ablation of endophilin A1 in CA1 recapitulates the behavioral phenotypes of pan-neural KO mice, and to avoid disruption of endophilin A1 function during early neurodevelopment, first we generated floxed *EEN1* alleles (*EEN1*^fl/fl^) by mating the KO first mice to FLPeR (flipper) mice (**Figure 1B**). We then applied bilateral stereotaxic injections of adeno-associated viral vectors encoding both enhanced GFP and the Cre recombinase (AAV-GFP-2A-Cre) or only EGFP (AAV-GFP) under the CaMKIIα promoter into hippocampal CA1 regions of 8-week-old *EEN1*^fl/fl^ mice (**Figure 2C**). Immunostaining of brain sections from mice 21 days after injection indicated that endophilin A1 expression was dramatically reduced in AAV-GFP-2A-Cre-infected pyramidal neurons in CA1 (**Figure 2D**).

Next we examined hippocampus-associated memory of mice 21 days after viral injection. Based on the results obtained from the Morris water maze test on WT, HET, and KO mice, we shortened the training phase to 8 days and tested the memory retention of animals with probe trial for 5 days and memory recall 1 month after training. Compared with AAV-GFP-injected mice, AAV-GFP-2A-Cre-injected mice were slightly retarded in learning the position of the hidden platform (**Figures 2E–H**). They also exhibited rapid forgetting of platform position in the probe trial (**Figures 2I,J**). Moreover, they exhibited defect to recall the platform location after training once again (**Figures 2I,K,L**). Further, ablation of endophilin A1 in the CA1 region also caused impairment of contextual fear memory. Compared with AAV-GFP-injected mice, AAV-GFP-2A-Cre-injected mice displayed lower levels of freezing behavior 72 h after training (**Figures 2M,N**). Collectively these data indicate that endophilin A1 in the hippocampal CA1 region is required for the retention of spatial and contextual fear memory in mature animals.

### Expression of Endophilin A1 in CA1 of Mature KO Mouse Brain Is Sufficient for Restoration of Spatial and Contextual Fear Memory

**FIGURE 3:**
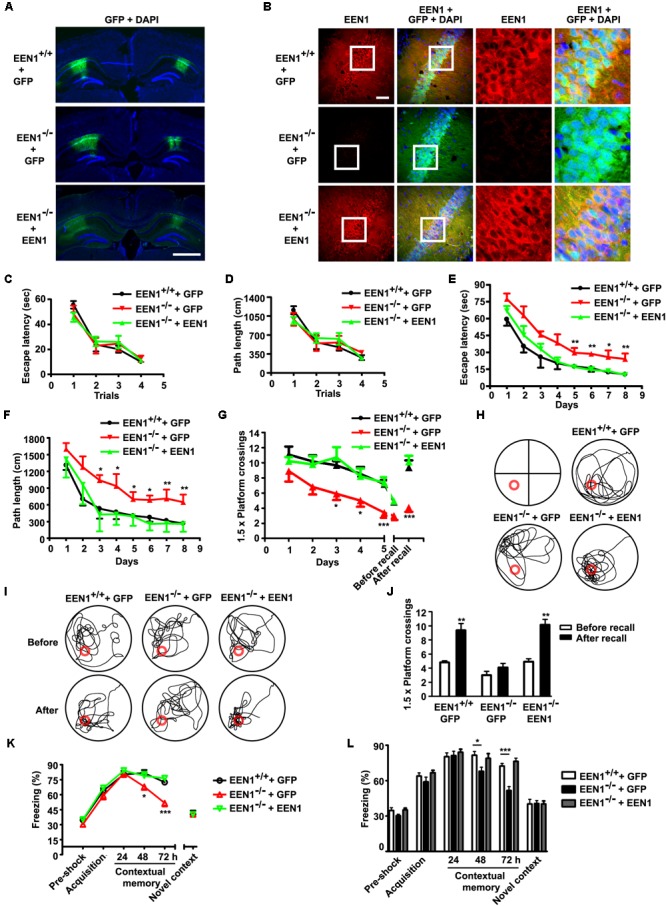
EEN1 overexpression in hippocampal CA1 of adult KO mice restores long-term spatial and contextual fear memory. **(A)** AAV virus was stereotaxically injected into the CA1 regions of *EEN1^-/-^* mice to express GFP alone or EEN1 and GFP. Shown are confocal images of GFP signal and DAPI labeling of nuclei 21 days after viral injection. Scale bar, 1 mm. **(B)** Immunofluorescence staining of EEN1 in CA1 neurons of brain slices 21 days after injection of AAV virus into the CA1 region of *EEN1^-/-^* mice. Right panels are magnification of the boxed areas. Scale bar, 100 μm. **(C–J)** The Morris water maze test. Shown are escape latency or traveled distance before escaping to the platform in the visible-platform training **(C** and **D)**, escape latency and traveled distance before escaping to the platform in the invisible-platform training **(E** and **F)**, number of crossing with the 1.5× platform area and the swim trace 5 days after training in probe test **(G** and **H)**, the swim trace and recall ability following training once again 1 month after training **(I** and **J)**. Data represent mean ± SEM (11 *EEN1^+/+^* + GFP, 10 *EEN1^-/-^* + GFP, 14 *EEN1^-/-^* + EEN1), ^∗^*p* < 0.05, ^∗∗^*p* < 0.01, ^∗∗∗^*p* < 0.001. **(K** and **L)** Restoration of freezing behavior in EEN1-overexpressed *EEN1^-/-^* mice, compared with control mice. Data represent mean ± SEM (11 *EEN1^+/+^* + GFP, 10 *EEN1^-/-^* + GFP, 14 *EEN1^-/-^* + EEN1), ^∗^*p* < 0.05, ^∗∗∗^*p* < 0.001.

To determine whether the memory impairment in *EEN1^-/-^* mice is irreversible or can be reversed by expression of endophilin A1 in adult brain, we injected AAV vectors coexpressing endophilin A1 and GFP (AAV-GFP-2A-EEN1) or GFP only into bilateral hippocampal CA1 regions of 8-week-old endophilin A1 KO mice (**Figure 3A**). Immunostaining of brain sections verified endophilin A1 expression in neurons infected with AAV-GFP-2A-EEN1 (**Figure 3B**).

The hippocampus-associated memory of mice was assayed 21 days after viral injection. All injected mice spent a similar time and traveled similar swimming distances before escaping onto the visible platform in the water maze test (**Figures 3C,D**). The performance of KO mice injected with AAV-GFP-2A-EEN1 was similar to that of WT mice in both the training phase and the probe trial (**Figures 3E–H**). Moreover, memory recall of the platform position exhibited by AAV-GFP-2A-EEN1-injected KO mice was indistinguishable from that by WT mice (**Figures 3G,I,J**). Consistently, compared with WT and AAV-GFP-injected KO mice, the AAV-GFP-2A-EEN1-injected KO mice did not exhibit any deficits in contextual fear memory (**Figures 3K,L**). Together, these data indicate that expression of endophilin A1 in the hippocampal CA1 region of mature brain is sufficient to rescue the memory deficits exhibited in *EEN1*^-^*^/^*^-^ mice.

### Loss of Endophilin A1 Impairs Postsynaptic Function and Long-Term Potentiation of Hippocampal CA1 Pyramidal Cells

**FIGURE 4:**
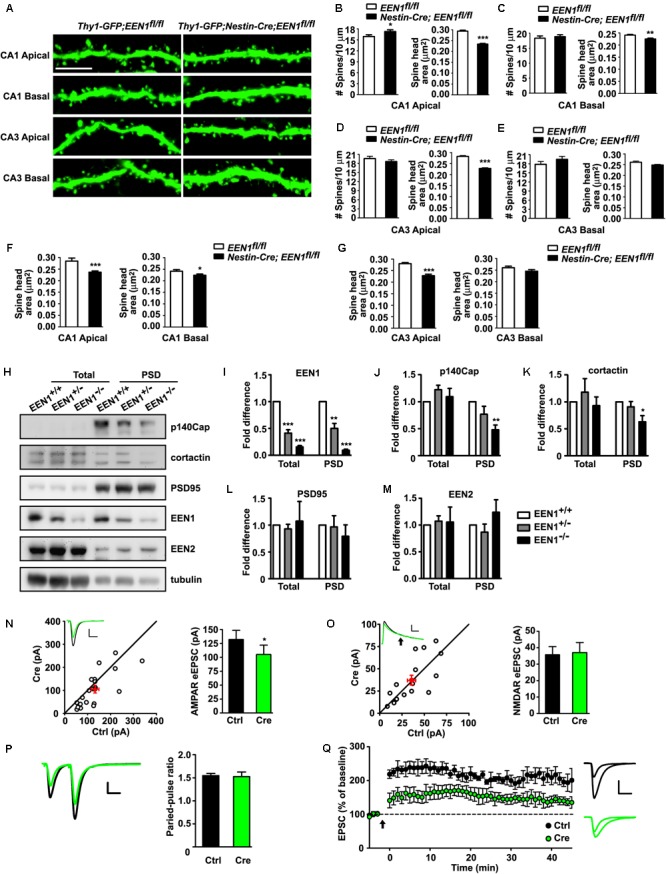
Morphological and functional alterations of EEN1-deficient hippocampal neurons. **(A)** Confocal micrographs showing spines on GFP-positive apical or basal dendrites of pyramidal cells in hippocampal CA1 or CA3 regions of 10-week-old *Thy1-GFP;EEN1^fl/fl^* and *Thy1-GFP;nestin-Cre;EEN1^fl/fl^* mice. Scale bar, 5 μm. **(B–E)** Quantification of spine density or spine head area in **A** (CA1 apical/basal: 42/34 cells, 2691/3240 spines, total length of dendrites >1500 μm and CA3 apical/basal: 32/31 cells, 2742/2258 spines, total length of dendrites >1000 μm for *Thy1-GFP;EEN1^fl/fl^*. CA1 apical/basal: 44/37 cells, 3666/2282 spines, total length of dendrites >1200 μm, and CA3 apical/basal: 39/32 cells, 3998/3002 spines, total length of dendrites >1500 μm for *Thy1-GFP;Nestin-Cre;EEN1^fl/fl^*). Data represent mean ± SEM, ^∗^*p* < 0.05, ^∗∗^*p* < 0.01, ^∗∗∗^*p* < 0.001. **(F–G)** Shown are the average of average spine head area of each CA1 and CA3 pyramidal cells, respectively. Data represent mean ± SEM, *n* = 31–44, ^∗^*p* < 0.05, ^∗∗∗^*p* < 0.001. **(H)** Immunoblotting of indicated proteins in homogenates (total) and PSD fractions from hippocampi of *EEN1^+/+^, EEN1^+/-^*, and *EEN1^-/-^* mice. **(I–M)** Quantification of protein levels in **H**, normalized to levels of *EEN1^+/+^* mice. Data represent mean ± SEM, *N* = 4, ^∗^*p* < 0.05, ^∗∗^*p* < 0.01, ^∗∗∗^*p* < 0.001. **(N)** Dual recording analysis of AMPAR-mediated synaptic responses. Scatter plots show amplitudes of AMPAR-eEPSCs for single pairs (open circles) and mean ± SEM (filled circle). The current amplitudes of infected neurons (Cre) were plotted on the ordinate and those of the control neurons (Ctrl) were plotted on the abscissa. Inset shows sample current traces from a pair of infected (green) and control (black) neurons. Scale bar, 100 pA and 20 ms. Bar graph shows mean ± SEM of AMPAR amplitudes represented in the scatter plots. Control, 132.1 ± 16.3 pA; Cre, 105.1 ± 16.8 pA, *n* = 20, ^∗^*p* = 0.030, paired *t*-test. **(O)** NMDAR-mediated eEPSC. Currents were recorded at +40 mV. Data were collected at 150 ms after electric stimulation (arrow), when the AMPAR-mediated EPSC had completely decayed. Scale bar, 50 pA and 50 ms. The NMDA eEPSCs were 35.7 ± 4.6 pA for control and 37.1 ± 5.7 pA for Cre-expressing neurons. *n* = 16, *p* = 0.74, paired *t*-test. **(P)** Paired-pulse recording of AMPAR eEPSCs. Two identical stimulus pulses were delivered in an interval of 50 ms and AMPAR eEPSCs were recorded at -70 mV. Left were sample traces of eEPSCs from a pair of infected and control neurons. Scale bar, 100 pA and 25 ms. The paired-pulse ratio (PPR) was the enhancement of the second eEPSC relative to the first eEPSC. Bar graph shows mean ± SEM of PPRs. Control, 1.55 ± 0.05; Cre, 1.53 ± 0.10, *n* = 10, *p* = 0.81, paired *t*-test. **(Q)** LTP was severely reduced in EEN1-deficient neurons. Relative amplitudes of AMPAR-eEPSCs (mean ± SEM) in control and Cre-expressing neurons before and after a whole-cell LTP-pairing protocol (arrow), Vm = 0 mV, 2 Hz SC stimulation for 90 s, normalized to average eEPSC amplitude prior to LTP induction. *n* = 10 decreased to 6 cells for control and *n* = 9 decreased to 6 cells for Cre-expressing neurons, respectively. Right shows sample traces of control and Cre before and 40 min after pairing. Sale bar: 100 pA and 20 ms. The potentiation ratio is significantly decreased in EEN1-deficient neurons 40 min after LTP induction, *p* = 0.020, *t*-test

To investigate changes in synaptic functions caused by ablation of endophilin A1 at the cellular level, first we examined neuronal morphology in the hippocampal CA1 and CA3 regions by crossing *Nestin-Cre^+/-^*; *EEN1^fl/fl^* mice with *Thy1-EGFP^+/-^*; *EEN1^fl/fl^* mice and imaging sparsely labeled neurons in brain sections by confocal microscopy (**Figure 4A**). Quantitative analysis indicated that there was a decrease in the size of spines of both the apical and basal dendrites of CA1, and apical dendrites of CA3 pyramidal cells in *EEN1*^-^*^/^*^-^ mice (**Figures 4B–D,F,G**). There was also a slight increase in spine density of CA1 apical dendrites (**Figure 4B**), possibly an *in vivo* compensation for the reduction in spine size. No statistically significant change in the number and size of spines was detected in CA3 basal dendrites (**Figures 4E,G**).

We also determined whether ablation of endophilin A1 causes changes in the levels of postsynaptic proteins in mouse hippocampi. Consistent with previous findings that endophilin A1 recruits p140Cap and cortactin, its downstream effectors, to dendritic spines (Yang et al., 2015), immunoblotting of the PSD fraction detected significant decreases in their amount in KO mice (**Figures 4H–K**). In agreement with the mild phenotype in neuronal morphology of CA1 and CA3 pyramidal cells, no significant changes in the postsynaptic levels of PSD95 in the whole hippocampus were detected (**Figures 4H,L**). Notably, the amount of endophilin A2, another member of the endophilin A family, in the PSD fraction was similar in *EEN1^+/+^, EEN1^+/-^*, and *EEN1^-/-^* mouse hippocampi (**Figures 4H,M**), indicating that loss of endophilin A1 did not cause its upregulation at the postsynaptic site. Unfortunately, we were unable to test expression of endophilin A3 because of lack of reliable antibodies for immunoblotting.

Spine size and synaptic strength are significantly correlated. As morphological changes in dendritic spines were detected in apical dendrites of CA1 pyramidal cells, which receive excitatory inputs from CA3, next we sought to determine whether synaptic transmission is impaired in *EEN1^-/-^* CA1 neurons by electrophysiological analysis. To investigate exclusively effect(s) of endophilin A1 ablation in postsynaptic neurons, we eliminated the *EEN1* gene in a small subset of CA1 neurons by injection of organotypic hippocampal slice culture from *EEN1^fl/fl^* mice with AAV-GFP-2A-Cre (Niu et al., 2017). By simultaneous recording of evoked excitatory postsynaptic currents (eEPSCs) on virus-infected and adjacent uninfected cells, we detected a decrease in AMPAR-mediated synaptic transmission in endophilin A1-deficient neurons (**Figure 4N**), whereas the NMDA-type glutamate receptor (NMDAR)-mediated eEPSCs and the paired-pulse ratio of AMPAR eEPSCs were unaffected (**Figures 4O,P**), indicating that the impairment of synaptic function was not due to reduction of presynaptic glutamate release. Further, we asked whether synaptic potentiation at SC-CA1 pathways was altered in the absence of endophilin A1. To this end, we injected the hippocampal CA1 region of *EEN1^fl/fl^* mice with AAV-GFP-2A-Cre at P0 and induced LTP in SC synapses by whole-cell recording of CA1 pyramidal cells in acute slices from virus-injected animals at P14-21 (Granger et al., 2013; Diaz-Alonso et al., 2017). In WT neurons, LTP induction caused a robust increase in EPSC that persisted throughout the 40-min recording period, whereas in AAV-GFP-2A-Cre-infected neurons the magnitude of LTP was significantly lower (**Figure 4Q**). Together these data indicate that both AMPAR-mediated basal transmission and LTP were impaired with removal of endophilin A1 in postsynaptic neurons.

### Endophilin A1, Not Endophilin A2 or A3, Is Required for the Structural and Functional Plasticity of Dendritic Spines Undergoing Synaptic Potentiation

**FIGURE 5:**
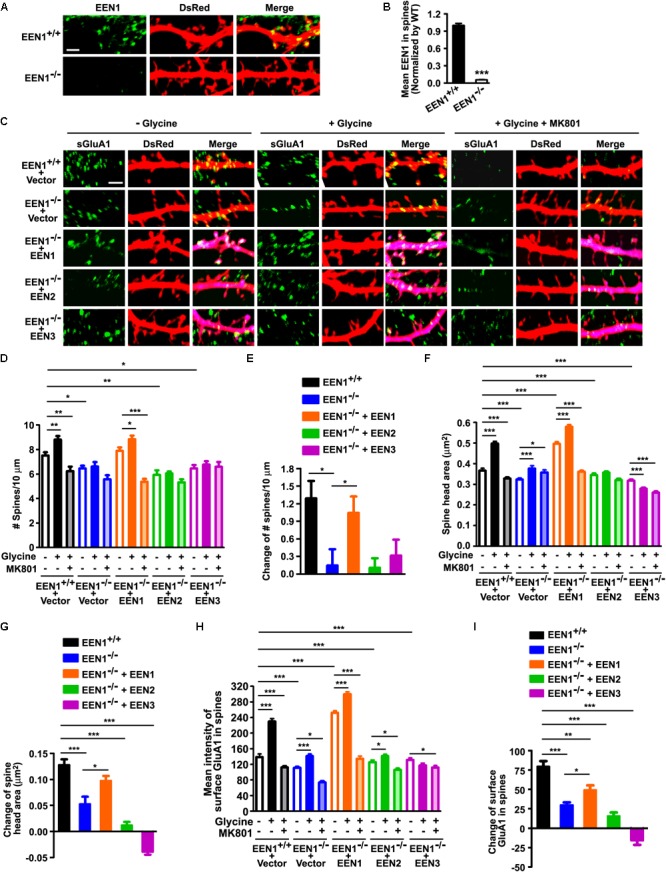
EEN1 is required for the structural and functional plasticity of dendritic spines. **(A)** Cultured *EEN1^+/+^* and *EEN1^-/-^* hippocampal neurons were transfected with pLL3.7.1 on DIV12-13 to express DsRed as volume marker, fixed and immunostained for EEN1 and DsRed on DIV19. Shown are representative confocal images of dendrites. **(B)** Quantification of EEN1 fluorescent signals in spines in **A**, normalized to levels of *EEN1^+/+^* neurons. Data represent mean ± SEM, *n* > 10 neurons, >600 spines per group, ^∗∗∗^*p* < 0.001. **(C)** Cultured *EEN1^+/+^* and *EEN1^-/-^* neurons co-transfected with DsRed expression construct and Flag vector, and *EEN1^-/-^* neurons co-transfected with constructs expressing DsRed- and Flag-tagged EEN1, EEN2, or EEN3 on DIV12-13 were treated with glycine to induce chemLTP with or without MK801 pretreatment on DIV18, and immunostained for surface GluA1, Flag, and DsRed. Shown are representative confocal images of dendrites. **(D)** Quantification of spine density in **C**. **(E)** Changes of spine density in **C**. **(F)** Quantification of spine head area in **C**. **(G)** Changes of spine head area in **C**. **(H)** Quantification of surface GluA1 levels in spines in **C**. **(I)** Changes of surface GluA1 levels in spines in **C**. Data represent mean ± SEM in **D–I**, *n* > 15 neurons per group, >850 spines per group, ^∗^*p* < 0.05, ^∗∗^*p* < 0.01, ^∗∗∗^*p* < 0.001. Scale bars, 2 μm.

Long-term potentiation is a form of long-term synaptic plasticity, the cellular correlate of learning and memory. At SC-CA1 synapses, LTP occurs when Ca^2+^ influx through the activated NMDARs in the postsynaptic membrane initiates downstream signaling cascades, leading to the structural and molecular remodeling of dendritic spines that eventually result in potentiation of AMPAR-mediated synaptic transmission (Herring and Nicoll, 2016). The finding that postsynaptic ablation of endophilin A1 in CA1 neurons impaired whole-cell LTP prompted us to investigate whether it functions in the structural and functional plasticity of dendritic spines. To this end, we used a well-characterized pharmacological approach (Park et al., 2006; Fortin et al., 2010) to chemically induce LTP (chemLTP) in mature hippocampal neurons in dissociated culture (**Figures 5A,B**). LTP induction by application of glycine led to a rapid increase in both the number and size of spines as well as surface levels of the AMPAR subunit GluA1 in *EEN1^+/+^* neurons, which was fully inhibited by the addition of MK801, an NMDAR antagonist (**Figures 5C,D,F,H**). In contrast, not only the activity-dependent increase in spine density was abolished in *EEN1^-/-^* cells, changes in spine morphology and GluA1 surface expression were also significantly inhibited (**Figures 5C–I**). The impairment in structural and functional plasticity was rescued by overexpression of endophilin A1 but not A2 or A3 (**Figures 5C–I**). Moreover, endophilin A1 overexpression failed to restore plasticity in MK801-treated *EEN1^-/-^* neurons (**Figures 5C,D,F,H**), indicating that endophilin A1 functions in NMDAR-mediated spine growth and synaptic potentiation. Notably, the absolute activity dependent increase in not only spine number but also spine size in endophilin A1-overexpressing *EEN1^-/-^* neurons was similar to those in *EEN1^+/+^* neurons (**Figures 5E,G**). Since overexpression of endophilin A1 caused enlargement of spines in steady state *EEN1^-/-^* neurons (**Figure 5F**), this result is consistent with previous findings that chemLTP induction causes similar modifications in small and large spines (Kopec et al., 2006). Taken together, these data indicate that endophilin A1, not endophilin A2 or A3, is specifically required for NMDAR-mediated synaptic potentiation of dendritic spines.

### Endophilin A1 Promotes Actin Polymerization in Dendritic Spines of Hippocampal Neurons Undergoing Synaptic Potentiation

**FIGURE 6:**
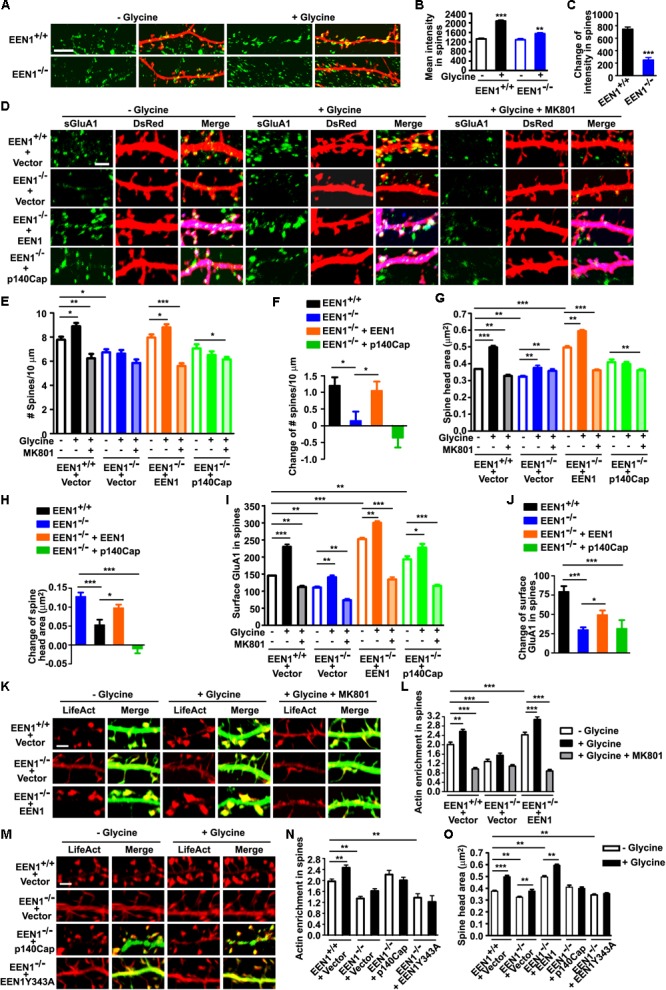
EEN1 promotes actin polymerization in spines undergoing synaptic potentiation. **(A)** Cultured *EEN1^+/+^* and *EEN1^-/-^* hippocampal neurons transfected with construct expressing DsRed on DIV12-13 were treated with glycine to induce LTP on DIV18, followed by immunostaining for p140Cap and DsRed. **(B)** Quantification of p140Cap mean intensity in spines in **A**. **(C)** Changes of p140Cap mean intensity in spines in **A**. Data represent mean ± SEM, *n* > 10 neurons, >500 spines per group, ^∗∗^*p* < 0.01, ^∗∗∗^*p* < 0.001. **(D)** Cultured *EEN1^+/+^* and *EEN1^-/-^* hippocampal neurons co-transfected with DsRed construct and Flag vector, and *EEN1^-/-^* neurons co-transfected with constructs expressing DsRed- and Flag-tagged EEN1 or p140Cap on DIV12-13 were treated with glycine to induce LTP with or without MK801 pretreatment on DIV18, followed by immunostaining for surface GluA1, Flag, and DsRed. Shown are representative confocal images of dendrites. **(E)** Quantification of spine density in **D**. **(F)** Changes of spine density in **D**. **(G)** Quantification of spine head area in **D**. **(H)** Changes of spine head area in **D**. **(I)** Quantification of surface GluA1 levels in spines in **D**. **(J)** Changes of surface GluA1 levels in spines in **D**. Data represent mean ± SEM in **E–J**, *n* > 15 neurons, >850 spines per group, ^∗^*p* < 0.05, ^∗∗^*p* < 0.01, ^∗∗∗^*p* < 0.001. **(K)** *EEN1^+/+^* neurons co-transfected with LifeAct–mCherry and GFP constructs, and *EEN1^-/-^* neurons co-transfected with LifeAct–mCherry and GFP or EEN1-GFP constructs on DIV12-13 were treated with glycine with orwithout MK801 pretreatment on DIV18, followed by immunostaining with antibodies against GFP and mCherry. Shown are representative confocal images of dendrites. **(L)** Quantification of actin enrichment in dendritic spines in **K**. Data represent mean ± SEM, *n* > 12 neurons, >700 spines per group, ^∗∗^*p* < 0.01,^∗∗∗^*p* < 0.001. **(M)** *EEN1^+/+^* and *EEN1^-/-^* neurons co-transfected with LifeAct–mCherry construct and Flag vector, and *EEN1^-/-^* neurons co-transfected with LifeAct–mCherry and Flag-tagged p140Cap or EEN1 Y343A constructs on DIV12-13 were treated with glycine on DIV18, followed by immunostaining with antibodies against mCherry and Flag. Shown are representative confocal images of dendrites. **(N)** Quantification of actin enrichment in dendritic spines in **M**. **(O)** Quantification of spine head area in **M**. Data represent mean ± SEM in **N** and **O**, *n* > 10 neurons, >500 spines per group, ^∗∗^*p* < 0.01, ^∗∗∗^*p* < 0.001. Scale bars, 5 μm in A and 2 μm in **D, K**, and **M**

During early stages of synaptic development, endophilin A1 contributes to dendritic spine morphogenesis and stabilization by recruiting p140Cap to spines to promote actin polymerization (Yang et al., 2015). Recent studies show that p140Cap regulates synaptic plasticity through Src-mediated and Citron-N-mediated actin reorganization (Repetto et al., 2014). To explore the mechanistic role of endophilin A1 in synaptic plasticity of postsynaptic neurons, first we asked whether recruitment of p140Cap to dendritic spines by endophilin A1 is regulated by neural activity. Indeed, an increase in p140Cap signal intensity in dendritic spines was detected in glycine-treated *EEN1^+/+^* neurons, which was attenuated in *EEN1^-/-^* neurons (**Figures 6A–C**).

Next we asked whether the endophilin A1-p140Cap pathway contributes to the increases in spine number and size and postsynaptic surface expression of GluA1 during chemLTP (**Figure 6D**). Intriguingly, although overexpression of endophilin A1 restored both structural and functional plasticity in *EEN1^-/-^* neurons, overexpression of p140Cap failed to ameliorate the defects in morphological changes of spines and upregulation of the postsynaptic GluA1 levels (**Figures 6D–J**). Since p140Cap is a downstream effector of endophilin A1, these data suggest that mechanism(s) other than the endophilin A1-p140Cap interaction are required for endophilin A1-mediated synaptic plasticity of spines. Alternatively, the interaction might be spatiotemporally regulated by activity-dependent signals upstream of endophilin A1. Nevertheless, since actin reorganization is crucial for spine plasticity (Hotulainen and Hoogenraad, 2010), next we asked whether endophilin A1 promotes actin polymerization in spines during chemLTP. To this end, we transfected neurons with constructs expressing EGFP and the F-actin probe LifeAct–mCherry. Quantification of the spine:shaft ratio of red fluorescence mean intensity revealed that indeed, the increase in F-actin content in spines was inhibited in glycine-treated *EEN1^-/-^* neurons, which was restored by overexpression of endophilin A1 (**Figures 6K,L**). Moreover, endophilin A1 overexpression failed to rescue activity-dependent F-actin accumulation in spines of MK801-treated *EEN1^-/-^* neurons (**Figures 6K,L**), indicating that endophilin A1 promotes actin polymerization via the NMDAR-mediated signaling pathway. Further, although overexpression of p140Cap fully rescued levels of F-actin in spines of steady state *EEN1^-/-^* neurons, overexpression of a p140Cap-binding deficient mutant of endophilin A1 (Y343A) (Yang et al., 2015) did not (**Figures 6M,N**). Intriguingly, neither p140Cap nor the endophilin A1 Y343A mutant could restore the increase in F-actin content or the size of spine head in glycine-treated *EEN1^-/-^* spines (**Figures 6M–O**), suggesting that the endophilin A1-p140Cap interaction is required not only for actin polymerization during spine morphogenesis and maturation, but also for spatiotemporal regulation of actin dynamics crucial for the activity-dependent morphological changes of spines. Collectively, these data indicate that endophilin A1 promotes actin polymerization in dendritic spines during synaptic potentiation.

## Discussion

In this study, we uncovered a postsynaptic role of endophilin A1 in synaptic plasticity distinct from that of endophilin A2 and A3. Specifically, endophilin A1 is required for the physical enlargement and upregulation of AMPAR expression in the postsynaptic membrane of dendritic spines during synaptic potentiation, whereas previous studies have indicated that endophilin A2 and A3 cooperate with the immediate early protein Arc/Arg3.1 to downregulate surface AMPARs by accelerating their endocytosis (Chowdhury et al., 2006). In agreement with its function in synaptic plasticity, KO of endophilin A1 in mouse brain causes deficits in spatial and contextual fear memory, which can be rescued by its overexpression in the hippocampal CA1 region. Whether or not endophilin A1 is also involved in higher brain function(s) that requires brain areas other than the hippocampus remains to be determined.

Endophilin A1 is highly expressed in the CA1 and CA3 regions of the hippocampus. Notably, ablation of endophilin A1 in the hippocampal CA1 region of mature brain is sufficient to cause phenotypes in spatial and contextual fear memory similar to those of pan-neural KO mice. Conversely, expression of endophilin A1 in CA1 of mature brain fully rescues the memory deficits of KO mice. Given that the CA2 subfield is essential for social memory, but is not critical for spatial and contextual memory (Hitti and Siegelbaum, 2014; Mankin et al., 2015), the region-specific expression of endophilin A1 might explain the learning and memory deficits of the KO mice, as both spatial and contextual fear memories involve the CA3–CA1 pathway.

Notably, there is only one endophilin A in *Drosophila melanogaster* and *Caenorhabditis elegans*. A role for endophilin A in synaptic vesicle recycling has been established across species (Ringstad et al., 1999; Gad et al., 2000; Verstreken et al., 2002; Schuske et al., 2003). Most recent studies reveal that endophilin A also functions in neuronal activity and stress-induced macroautophagy at presynaptic terminals of NMJ in *Drosophila*, which mediates protein turnover and is crucial for neuronal homeostasis and survival (Soukup et al., 2016). Endophilin A1 phosphorylated at the Ser75 residue by the kinase LRRK2 (Matta et al., 2012) promotes autophagosome formation by creating highly curved membrane zones in preautophagosomes that serves as docking sites for autophagic factors (Soukup et al., 2016). In mammalian cells, all three endophilin As interact with the E3 ubiquitin ligase FBXO32 that is involved in protein homeostasis, and both endophilin A1 and A2 are needed for autophagosome formation in mouse neurons (Murdoch et al., 2016). Moreover, recent studies also indicate that endophilin As regulate endosomal sorting and trafficking of the BDNF–TrkB neurotrophic signal complex to mediate dendrite development and survival of hippocampal neurons (Fu et al., 2011; Burk et al., 2017). The endophilin A DKO and TKO mice but not single KO mice show ataxia and motor impairment caused by neurodegeneration in the brain (Murdoch et al., 2016), suggesting functional redundancy of endophilin A family members in autophagosome formation and endosomal sorting.

In higher eukaryotes, however, the biological functions of endophilin A family members at the postsynaptic site are diverse. Removal of endophilin A in *Drosophila* causes a reduction in the frequency but not amplitude of miniature excitatory junctional potentials (mEJPs) at the NMJ (Verstreken et al., 2002). In contrast, both the frequency and amplitude of mEPSCs of dissociated cultured hippocampal neurons from the TKO mice are lower than the WT animals (Milosevic et al., 2011). As a decrease in the amplitude of mEPSCs indicates impaired synaptic response to neurotransmitter release from a single vesicle, together these findings suggest that the postsynaptic function(s) of endophilin A is required to maintain synaptic function in mammalian neurons. Consistent with previous findings that transient knockdown of endophilin A1 expression in cultured hippocampal neurons causes a decrease in the frequency of mEPSCs (Yang et al., 2015), AMPAR-mediated basal transmission is impaired in endophilin A1-deficient CA1 neurons. Moreover, ablation of endophilin A1 in CA1 neurons also causes impairment in LTP. Notably, the impaired structural and functional plasticity of dendritic spines of endophilin A1-deficient neurons cannot be rescued by other endophilin A family members, indicating that its role in synaptic plasticity is distinct from those of A2 and A3.

What is the mechanistic role(s) of endophilin A1 in synaptic plasticity? Given that it facilitates actin polymerization by recruiting p140Cap and cortactin to dendritic spines during spine morphogenesis and maturation (Yang et al., 2015), it is conceivable that endophilin A1 also promotes actin polymerization required for formation of new spines and increase in the size of existing spines during synaptic potentiation. Intriguingly, overexpression of p140Cap, its downstream effector, does not rescue the phenotypes of endophilin A1 KO neurons during chemLTP. As the turnover rates and locations of distinct F-actin pools in single dendritic spines are dynamically regulated during synaptic plasticity (Honkura et al., 2008; Frost et al., 2010), these findings suggest that actin polymerization promoted by the p140Cap pathway is not sufficient for restoration of actin cytoskeleton remodeling in spines, and that other regulatory factor(s) acts via endophilin A1 to achieve the spatiotemporal control of molecular events required for the growth and synaptic potentiation of dendritic spines. A recent study reports that calmodulin binds to the N-BAR domains of endophilin A1 and A2 *in vitro* and promotes the membrane tubulation activity of endophilin A2 in COS7 cells (Myers et al., 2016). Ca^2+^/calmodulin-dependent activation of calmodulin-dependent protein kinase (CaMKII) plays a central role for the induction of LTP (Herring and Nicoll, 2016). Given that the Ca^2+^-calmodulin–CaMKII pathway controls signaling cascades that regulate both branched actin polymerization and receptor trafficking at synapses, and that endophilin A1 also functions in promoting actin polymerization in spines, it will be of great interest to explore whether and how endophilin A1 functions downstream of these two master regulators of intracellular signaling during synaptic plasticity.

## Author Contributions

YY, YSS, and J-JL designed the experiments. YY, JC, ZG, SD, XD, SZ, and CY performed the experiments. YY, JC, ZG, and J-JL analyzed the data. YY, YSS, and J-JL wrote the paper.

## Conflict of Interest Statement

The authors declare that the research was conducted in the absence of any commercial or financial relationships that could be construed as a potential conflict of interest.
